# ATPase family AAA domain-containing protein 2 (ATAD2): From an epigenetic modulator to cancer therapeutic target

**DOI:** 10.7150/thno.78840

**Published:** 2023-01-01

**Authors:** Jiahui Fu, Jin Zhang, Xiya Chen, Zhiying Liu, Xuetao Yang, Zhendan He, Yue Hao, Bo Liu, Dahong Yao

**Affiliations:** 1 School of Pharmaceutical Sciences, Shenzhen Technology University, Shenzhen, 518118, China.; 2 State Key Laboratory of Biotherapy and Cancer Center, West China Hospital, Sichuan University, Chengdu 610041, China.; 3 School of Pharmaceutical Sciences, Medical School, Shenzhen University, Shenzhen 518060, China.

**Keywords:** ATPase family AAA domain-containing protein 2 (ATAD2), Epigenetic modification, Therapeutic target, Small-molecule inhibitor, Cancer therapy, Anticancer drug

## Abstract

ATPase family AAA domain-containing protein 2 (ATAD2) has been widely reported to be a new emerging oncogene that is closely associated with epigenetic modifications in human cancers. As a coactivator of transcription factors, ATAD2 can participate in epigenetic modifications and regulate the expression of downstream oncogenes or tumor suppressors, which may be supported by the enhancer of zeste homologue 2. Moreover, the dominant structure (AAA + ATPase and bromine domains) can make ATAD2 a potential therapeutic target in cancer, and some relevant small-molecule inhibitors, such as GSK8814 and AZ13824374, have also been discovered. Thus, in this review, we focus on summarizing the structural features and biological functions of ATAD2 from an epigenetic modulator to a cancer therapeutic target, and further discuss the existing small-molecule inhibitors targeting ATAD2 to improve potential cancer therapy. Together, these inspiring findings would shed new light on ATAD2 as a promising druggable target in cancer and provide a clue on the development of candidate anticancer drugs.

## 1. Introduction

**Fig 1:**
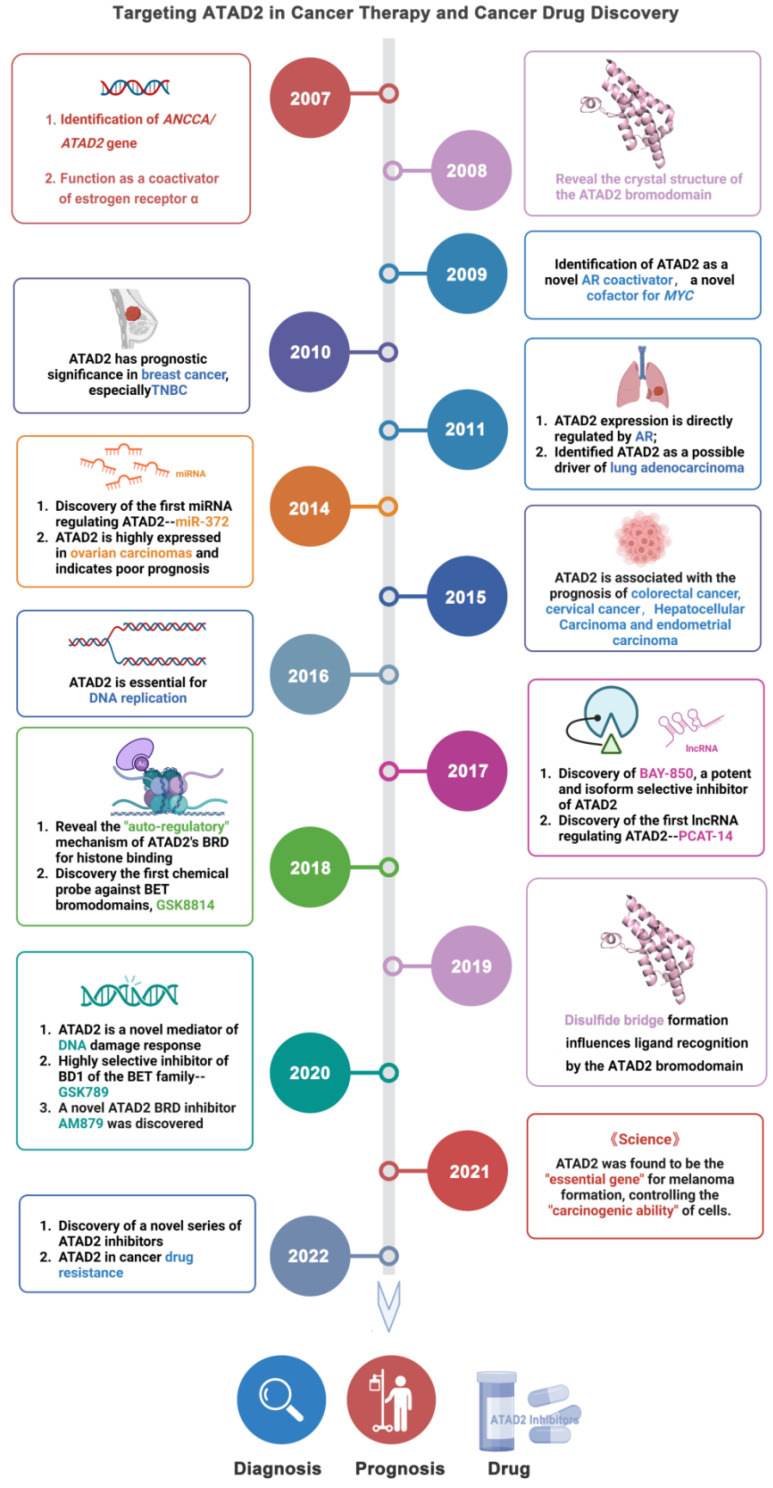
Timeline illustrating the development of ATAD2 as a potential candidate biomarker and druggable target in cancer therapy.

AAA ATPase proteins, also known as ATPase proteins, are composed of approximately 200 amino acids and gradually oligomerize into hexamers. This enzyme structure contains ATP binding sites, which can catalyze the hydrolysis of ATP to ADP and phosphate ions with releasing energy 1. Notably, these energies are essential for organisms to maintain biological functions, such as DNA replication, priming, and remodeling; protein synthesis, modification, and degradation; and transport of nutrients and metabolites 2. With in-depth research, increasing ATPase family members have been reported 3, 4. Of them, given the close relationship between ATAD2 and cancer, which has become an increasingly recognized research hotspot 5, 6 **(Figure 1).**

As an epigenetic regulator, ATAD2 is first known for its role in histone modification. In 2007, *ANCCA*/ATAD2 was first confirmed as a coactivator of estrogen receptor α (ERα). ANCCA binding and hydrolyzing ATP is critical for co-regulating the recruitment of cAMP-responsive element-binding protein (CBP/p300) and histone hyperacetylation on ER target chromatin, providing new insights into the potential mechanisms of *ANCCA* in breast cancer development 7. ATAD2 has since been shown to also act as a coactivator of other transcription factors, including c-Myc and the E2F transcription factor 1 (E2F1), E2F transcription factor 2 (E2F2), and E2F transcription factor 3 (E2F3) proteins 8, 9. In the following decade, studies on ATAD2 became more comprehensive and in-depth, from the upstream factors regulating ATAD2 protein expression (such as miR-372, lnc PACT-14) to the mechanism of action involved in the occurrence and development of breast cancer, lung adenocarcinoma, liver cancer and other diseases 10-13. In particular, ATAD2 plays an important role in histone modification of tumor cells 14, 15. For instance, in prostate cancer LNCaP cells, ATAD2 affects the recruitment of histone methylase mixed lineage protein-1 (MLL1) and RNA polymerase II during enhancer of zeste homologue 2 (*EZH2*) gene transcription. In addition, ATAD2 was confirmed to be closely related to the mRNA and protein levels of the oncogene, *EZH2* 16. EZH2, the main catalytic subunit of polycomb repressive complex 2, catalyzes the trimethylation modification of H3K27 through its SET domain to maintain the silencing state of downstream target genes. Notably, EZH2 is reported to be highly expressed in many human tumors, promoting tumorigenesis and malignant transformation 17, 18. In prostate cancer, ATAD2 can bind to the promoter of *EZH2* together with KDM8 and E2F1 to promote the transcription of EZH2. Therefore, the regulation of EZH2 expression by ATAD2 plays a significant role in the development of cancer 19.

More excitingly, recently it is reported that ATAD2 appears to be a necessary gene for melanoma formation in a melanoma zebrafish model, making ATAD2 a potential cancer therapeutic target. When ATAD2 is knocked out, the cells cannot become cancerous, despite numerous oncogenic and antioncogenic mutations. In contrast, the addition of ATAD2 allows the cells to regain their ability to become cancerous, meaning that ATAD2 is an important factor in melanoma formation 14. Admittedly, ATAD2 is indeed highly expressed in many cancers and is associated with poor prognosis in cancers including breast, colon and cervical cancer 20, 21. Over-expressed ATAD2 interacts with various transcription factors and chromatin-modifying proteins in cancer cells and promotes tumor growth by inducing the full expression of genes that promote cell proliferation and inhibit cell apoptosis 22.

Additionally, protein crystal structures of ATAD2 and targeted small-molecule compounds have been deciphered one by one 23-26. Structurally, the AAA + ATPase domain can mediate ATAD2 polymerization, and mutations in this region will lead to changes in its downstream cascade, such as the regulatory relationship between ATAD2 and ERα 7. Within the central region of ATAD2, in addition to a functional AAA + ATPase domain, there is also a bromine domain (BRD) near the C-terminus. BRD can specifically recognize peptide chains containing acetylated lysine residues, target ATAD2 protein to activate transcription regions, regulate chromatin structure, and recruit other transcription factors to participate in post-translational modifications of proteins 27. Currently, a series of compounds regulating BRD have emerged, opening up new prospects for anti-cancer drugs, such as JQ1, AZ13824374 and AM879 28-30.

Taken together, targeting ATAD2 is a promising strategy for cancer treatment. This review mainly focuses on ATAD2 from the protein structure, biological function, signaling pathway and other aspects to launch a detailed introduction. More importantly, we systematically summarize the design and discovery of small molecule inhibitors that regulate ATAD2, and the characteristics, advantages and disadvantages of these small molecules, which may provide a cornerstone for the development of ATAD2 target drugs for cancer therapy.

## 2. The structure of ATAD2

**Fig 2:**
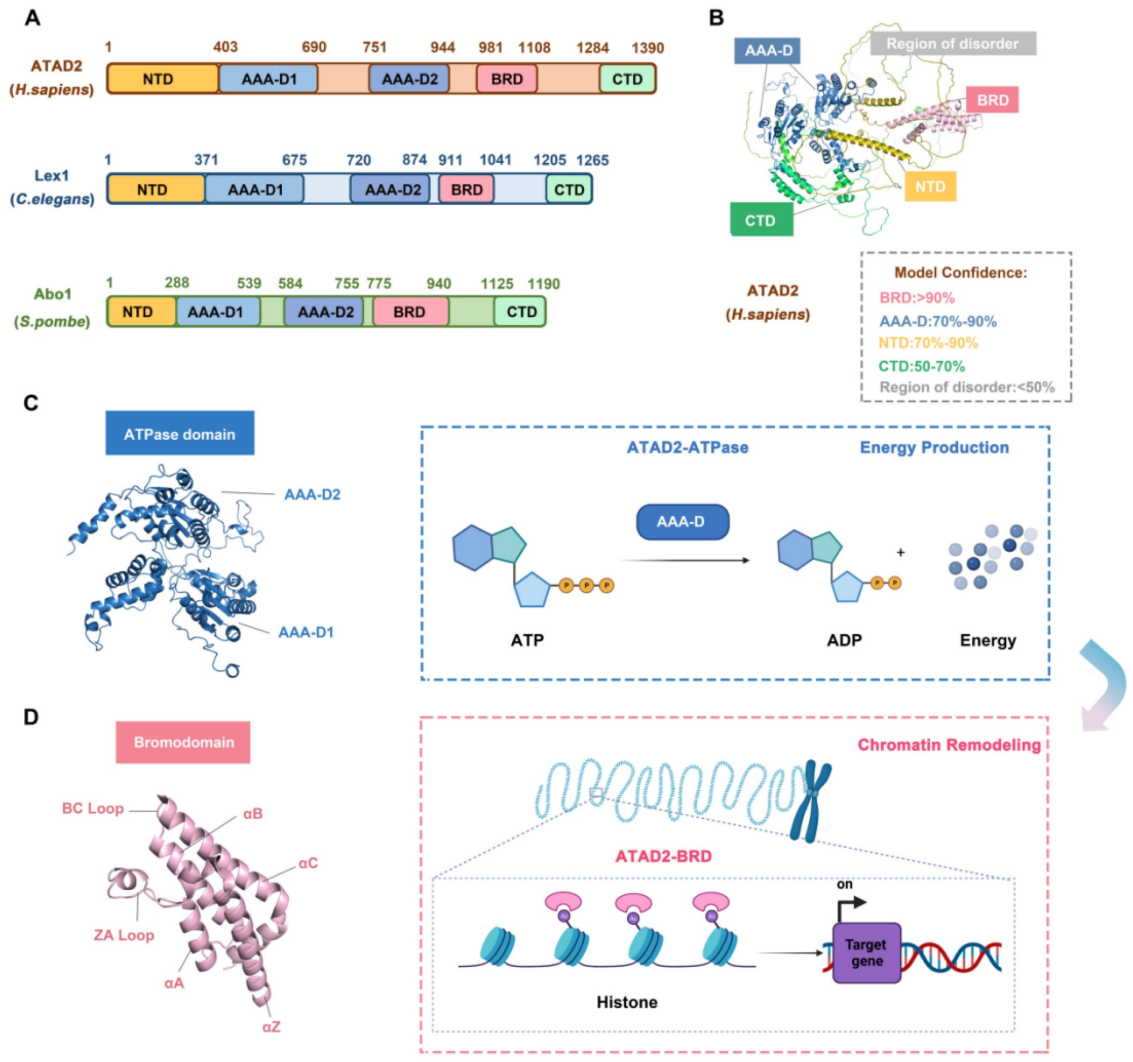
** The structure and functions of ATAD2 domains.** (A) The domain structure of human ATAD2, C. elegans Lex1, and S. pombe Abo1. NTD, N-terminal acidic domain; AAA-D, AAA+ ATPase domain; BRD, bromodomain; CTD, C-terminal domain. (B) The structure of ATAD2 predicted by AlphaGo 37 and the confidence levels for each domain are also shown. (C) The ATPase domain structure and main function of ATAD2. (D) The bromodomain structure and main function of ATAD2.

The N-terminal acidic domain (NTD, residues 1-403), bipartite domain (AAA-D1, residues 403-690) and AAA-D2 (residues 751-944), AAA + ATPase domain (residues 403-944), bromodomain (BRD, residues 981-1108) and C-terminal domain (CTD, residues 1264-1390), a total of 1390 amino acids, constitute ATAD2. Studies have proven that ATAD2 is evolutionarily conserved across multiple species. Abo1 of *S. pombe* and lex-1 of *C. elegans* share the same conserved domain feature with human ATAD2 but differ in the location of the residues in which the domains are located. For example, in *C. elegans* and *S. pombe*, the NTD residues are residues 1-371 and 1-288, respectively, and the BRD residues are residues 981-1108 and 755-940 31** (Figure 2A-B).**

The AAA + ATPase domain is near the NTD module of ATAD2 and consists of a double hexamer consisting of AAA-D1 and AAA-D2, and the former is structurally located on top of the latter. AAA-D1 contains conserved Walker A (GXXXXGKT/S) and B motifs, sensors 1 and 2, and an arginine finger, which is the other conserved domain of ATAD2. The AAA + ATPase domain mediates the oligomerization of proteins to achieve the function of BRD-ATAD2 proteins and is responsible for ATP binding and hydrolysis. Increasing studies have proven that the AAA + ATPase domain mutation will affect its function as an ERα coactivator and interfere with DNA replication 32 **(Figure 2C).**

BRD, which is a highly conserved chromatin reader domain, is vital for chromatin modification. Structurally, all BRDs have a unique secondary structure, a left-handed bundle structure of four α-helices (αZ, αA, αB, αC) connected through ZA (αZ-αA) and BC (αB-αC) loops. Functionally, BRD binds and recognizes the acetylated lysine of chromatin, which know as a “reader”, to stimulate the transcription of target genes 33. BRD-ATAD2 is essential for the interaction of ATAD2 with histones and ATAD2-mediated cancer processes 34. Of note, the BRD-ATAD2 has a unique tectonic fold, named the “BRD fold” or lysine acetylation (KAc) binding pocket. Within this KAc pocket, some highly conserved residues, such as Asn1064 residues, which are directly involved in mediating histone interactions, are arranged here. Thus, the heterogeneous flexibility of the KAc pocket may affect the binding of ATAD2 to ligands and biological function 35 **(Figure 2D).**

There is mutual communication between the AAA + ATPase domain and BRD-ATAD2, which is in harmony with the finely regulated functions of ATAD2. First, the ATPase domain utilizes ATP hydrolysis to make bromodomain accessible to acetylated histone tails. Second, multiple bromodomains help to better "capture" proteins. Importantly, when the protein is in a multimeric form or in a high molecular weight complex, the interaction between the AAA + ATPase domain and BRD-ATAD2 improves the binding efficiency of the acetylation site of histone H4 36.

Therefore, the structure of ATAD2 is the basis of its executive function, and the analysis and study of its structure provide the structural basis and the possibility of drug design for the development of anticancer drug candidates by targeting ATAD2.

## 3. ATAD2 in cancer

**Fig 3:**
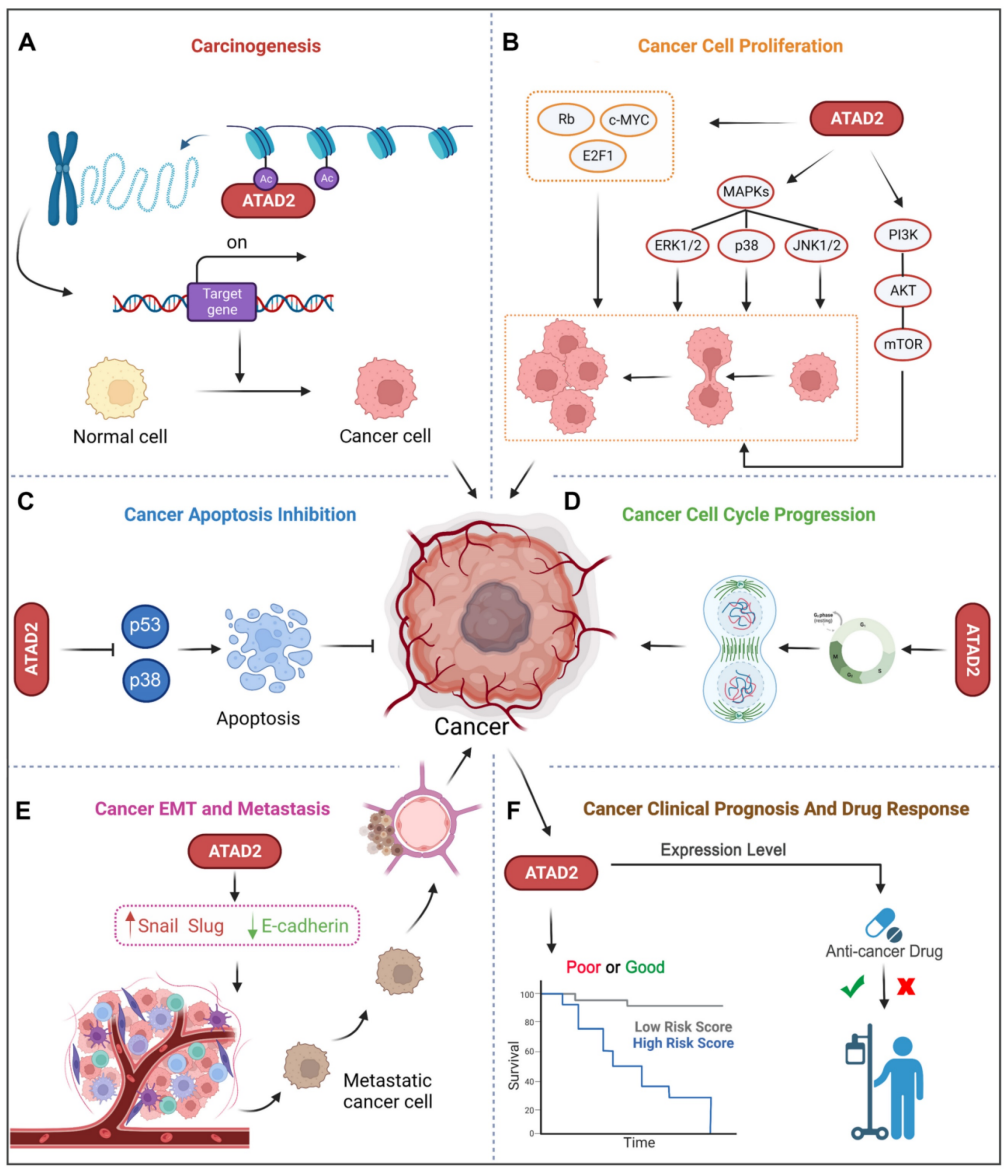
** ATAD2 in cancer.** ATAD2 is involved in multiple processes of cancer occurrence and development, which mainly include 6 major parts. (A) Promoting carcinogenesis by chromatin remodeling. (B) Facilitating uncontrollable proliferation of cancer cells. (C) Inhibiting cell apoptosis. (D) Accelerating the cancer cell cycle progression. (E) Promoting cancer EMT and metastasis. (F) Potential to predict cancer clinical prognosis and drug response of ATAD2.

Accumulating evidence has revealed that ATAD2 is involved in the carcinogenesis, proliferation, apoptosis and metastasis of various tumor cells. Importantly, ATAD2 is essential for tumorigenesis in melanoma 14, and overexpression of ATAD2 can facilitate the proliferation of tumor cells and impede apoptosis in a variety of malignancies **(Figure 3A-C)**. For example, overexpression of ATAD2 can activate the mitogen-activated protein kinase (MAPK) pathway to promote the proliferation of ovarian cancer cells 5. Furthermore, ATAD2 promotes tumor progression as a coactivator of the hormone-induced nuclear receptors ERα and androgen receptor (AR), E2Fs and c-Myc 38. In lung adenocarcinoma and gastric cancer, ATAD2 could promote cell proliferation via the phosphatidylinositol 3 kinase (PI3K) / AKT pathway and the retinoblastoma tumor suppressor protein (Rb)-E2F-cMyc signaling 39, 40. While in lung adenocarcinoma, the knockdown of ATAD2 affects glucose metabolism in cancer cells by regulating [18F] FDG uptake and lactate production through the AKT-GLUT1 / HK2 pathway 39. In addition, silencing ATAD2 also inhibits the growth and colony formation ability of esophageal squamous cell carcinoma (ESCC) cells, and significantly inhibits ESCC tumor growth* in vitro* 41.

Of note, abnormal regulation of the cell cycle is also a major feature of tumors, and knockdown of the ATAD2 gene leads to decreased expression of cyclin C and cyclin D1 in hepatocellular carcinoma, which is beneficial for cancer treatment 38. Similarly, the depletion of ATAD2 arrests the cell cycle in G1/S phase and triggers apoptosis through the Rb-E2F1 pathway 42. And silencing ATAD2 inhibits the proliferation of gastric cancer cells by reducing the expression of the key cell cycle regulator proteins cyclin D1, Rb, E2F1 and cyclin E, thereby arresting the cell cycle in the G1/S phase 40 **(Figure 3D)**.

As we know, metastasis and invasion are one of the malignant behaviors of tumor, and also an important factor leading to high mortality. Studies have shown that the loss of ATAD2 results in decreased protein expression of EMT to impede cancer cell migration and invasion 43. Silencing ATAD2 inhibited ESCC cell growth and lung metastasis *in vivo*, and the knockdown of ATAD2 inhibited ESCC migration and invasion *in vitro* 41. Additionally, the reduction in ATAD2 could induce a decrease in N-cadherin and Vimentin and an increase in E-cadherin, which is also beneficial for metastasis inhibition 44 **(Figure 3E)**.

In addition to the above-mentioned tumor characteristics, ATAD2 has also been used to evaluate the clinical prognosis of breast cancer (BC), liver cancer, GC and other cancers 39. Besides, ATAD2 overexpression was significantly associated with poorer overall survival, relapse-free survival, disease-free survival, and disease-specific survival, suggesting an indicative role for ATAD2 in the prognostic significance of human cancers 45. Some tumor biomarkers (B7-H4, PD-L1 and CMTM6) was notably correlated with ATAD2 in human oral squamous cell carcinoma (OSCC), and it is not surprising that high expression of ATAD2 indicates poor prognosis in OSCC 46. Furthermore, ATAD2 is also closely related to the cancer drug response, and deletion of ATAD2 has previously been indicated to induce cancer cell apoptosis, both in drug-sensitive and drug-resistant cancer 20. Remarkably, over-expression of ATAD2 was also significantly associated with tumor size, differentiation, and clinical lymph node metastasis stage and could be used as an independent prognostic factor in GC patients 40 **(Figure 3F)**. Hence, ATAD2 is a promising target for cancer therapy.

## 4. The molecular basis underlying the regulatory mechanisms of ATAD2

### 4.1 Upstream regulators

Considering the delicate and complex role of ATAD2 in cells, we first summarized its upstream regulators, including transcriptional regulation, post-transcriptional modification, and protein-protein interactions.

#### a. miRNAs

MicroRNAs (miRNAs), non-coding RNAs, have the capacity to modulate downstream gene expression by targeting the 3'UTR of target genes 47. miRNAs negatively regulate gene expression by RNA-induced silencing complex (RISC)-mediated mRNA processing or translational inhibition. Interestingly, studies have demonstrated that a number of miRNAs could regulate the development of different cancer cells by targeting ATAD2 48. For instance, miR-520f can restrain the increase in GC cells by targeting the regulation of ATAD2 49. MiR-200b-5p inhibits ovarian cancer cell proliferation and promotes apoptosis by targeting ATAD2 and regulating the PI3K/AKT signaling pathway 50. MiR-372 down-regulates the expression of ATAD2 to inhibit the growth, invasive and migratory capacity of liver cancer cells or renal cell carcinoma cells 13, 51. Over-expression of miR-186 can reduce the expression of ATAD2, inhibit the Hedgehog signaling pathway and ultimately inhibit the proliferation, migration, invasion and apoptosis of retinoblastoma cells 48. Besides, miR-302 increases the cisplatin sensitivity of ovarian cancer cells by inhibiting ATAD2 to hinder the invasiveness and EMT in cisplatin-resistant cells 44. In addition, miR-217 has also been reported to bind to the 3'UTR site, inhibit ATAD2 protein expression and induce apoptosis of pancreatic cancer cells 52. In summary, miRNAs could directly target and down-regulate the expression of ATAD2 to control cancer progression 53.

#### b. lncRNAs

Long noncoding RNAs (lncRNAs), with a length of more than 200 nucleotides, are non-translational RNAs that are involved in various cellular processes, including regulation of chromatin structure and transcriptional regulation. Importantly, lncRNAs act as sponges for miRNAs and signal the development of cancer by binding to target genes 47. PCAT-14 has been reported to promote the proliferation and invasion of cancer cells by inducing miR-372 methylation to increase ATAD2 expression, and then activating the hedgehog signaling pathway in hepatocellular carcinoma 54. Nuclear paraspeckle assembly transcript 1_2 (NEAT1_2), a common noncoding RNA subtype, can be used as a competitive endogenous RNA of miR-106B-5p to downregulate miR-106B-5p and increase the expression of ATAD2. Therefore, NEAT1_2 knockdown or silencing can inhibit the growth, migration and invasion of thyroid cancer cells 55. Moreover, MALAT1 promoted the expression of ATAD2 to accelerate cell proliferation, migration, invasion and EMT in Rb by sponging miR-655-3p 43. LncRNA CRNDE affects CRC cell progression by modulating the miR-126-5p/ATAD2 axis in CRC cells 56. Thus, lncRNAs also contribute significantly to the regulation of ATAD2 in cancer.

#### c. Transcription factors and proteins

ATAD2 acts as a cofactor to regulate genes with oncogenic functions together with certain transcription factors, such as c-Myc, E2F, androgen and estrogen receptors 42. Moreover, ATAD2 has also been identified as a coactivator of ERα, AR, E2Fs, and c-Myc for the promotion of tumor progression 38. Similarly, ATAD2 can be regulated by a variety of transcription factors or proteins. ATAD2 promoter contains seven potential E2F DNA-binding sites, and E2F1 binds to the regulatory region of the ATAD2 gene to activate its gene transcription 57. Notably, E2F1 and AR directly target ATAD2 to enhance ATAD2 gene expression at both the mRNA and protein levels 58. In addition to ERα, AR, and E2Fs, ATAD2 is also a coactivator of c-Myc, enhancing its transcriptional activity 39. Meanwhile, ATAD2 is a hypoxia-responsive and HIF1α-regulated gene, and HIF1α acts as a transcription factor to mediate the upregulation of ATAD2 in hypoxic SCC by binding to the ATAD2 promoter 59. Recently, a study found that the transcription factor MYBL2 can bind to the promoter of ATAD2 to promote the expression of ATAD2 to trigger ovarian cancer cell proliferation. Interestingly, when ATAD2 is knocked out, the stability of MYBL2 is also severely reduced as a fed back 60. Interestingly, ATAD2 can also be directly or indirectly regulated by a variety of proteins. For instance, KDM8 / JMJD5, a histone lysine demethylase/dioxygenase, directly targets ATAD2 to maintain cell survival via AR activation of EZH2 in prostate cancer 19. Besides, TRIM25 is an oncogenic ubiquitin E3 ligase that feedback regulates ATAD2 expression in a dose-dependent and transcription-independent manner to promote CRC development 61. Methyltransferase-like 3 (METTL3), a reported m6A “writer”, was found to promote the growth and invasion of osteosarcoma by enhancing the expression of ATAD2, but the regulatory mechanism is unclear 6. Additionally, the endoplasmic reticulum-associated degradation protein Derlin-1 can upregulate the expression of ATAD2, but the mechanism is also unclear 62. Notably, ATAD2 recently proved to be a direct target gene of YAP1, whose expression can be enhanced by YAP1 activation. Surprisingly but intriguingly, recruitment of BRD4, TEAD4 and YAP1/TAZ at ATAD2 promoter is interdependent, which can fine-regulate the expression intensity of ATAD2 together 63.

Therefore, ATAD2 can be fine-tuning by different regulators to cope with a variety of life processes in cancer, which also provides a theoretical basis for targeting ATAD2 for cancer treatment.

### 4.2 ATAD2-modulated cancer-associated signaling pathways

As an important epigenetic target, ATAD2 can regulate the expression of various cancer-related genes and participate in all aspects of cancer progression. In this section, we mainly elaborate on the cancer pathways regulated by ATAD2 and further clarify the molecular basis of ATAD2 as a promising candidate cancer target.

#### 4.2.1 ATAD2 in Chromatin Remodeling

As an important epigenetic target, ATAD2 is best known for its role in chromatin remodeling. Of note, chromatin remodeling has been a hot topic in epigenetic research in recent years, and it is also an important issue in cancer biology 66, 67. Mechanistically, ATAD2 directly binds to chromatin via histone acetylation identification, leading to an increase in chromatin accessibility and histone dynamics, which provide sufficient conditions for the appropriate activity of the highly expressed gene fraction. Additionally, ATAD2 acts as a regulator of chromatin dynamics to maintain specific gene expression programs through histone acetylation-directed chromatin opening 68. Besides, a structural study of ATAD2 found that BRD-ATAD2 can specifically direct proteins to acetylated histones, and its AAA + ATPase domain can act as a molecular motor in the process of acetylated histones. In this process, the acidic N-terminal region of ATAD2 binds tightly but nonspecifically to chromatin, then the AAA + ATPase domain promotes nucleosome expulsion, and the BRD read the acetylated lysines on histones 69. Intriguingly, recently a study has found that the D/E repeats sequence of ATAD2 is closely related to its chromatin binding capacity, and the chromatin binding capacity of ATAD2 with D/E repeats knockout is greatly reduced 70, as is the corresponding chromatin remodeling capacity. Undoubtedly, ATAD2 could regulate cancer progression by controlling chromatin remodeling. For instance, a recent study revealed that ATAD2 could promote the formation of melanoma phenotypes through chromatin remodeling. In detail, ATAD2 led to a significant increase in chromatin accessibility and formed a complex with SOX10 that promoted the expression of MAPK-related genes 14. In hepatocellular carcinoma, clinical value and biological function comprehensive bioinformatics analysis showed that ATAD2 plays an important role in carcinogenesis by interfering with the interaction between chromatin proteins and DNA 38. In short, ATAD2 can indeed promote cancer progression through chromatin remodeling.

#### 4.2.2 ATAD2 in DNA Replication, DNA Damage and Repair

In addition to chromatin remodeling, ATAD2 can also participate in the regulation of DNA replication, DNA damage and repair in cancer. DNA replication occurs in the S phase while ATAD2 is always highly expressed in this phase and directly regulates DNA replication. During this process, ATAD2 is recruited to DNA replication sites through interaction with the di-acetylation mark and binds to newly synthesized histones, which may assist in heterochromatin compression 32. Furthermore, the newly synthesized histone H4 was incorporated into chromatin during DNA replication at AcK5 and AcK12 sites. Overexpression of ATAD2 can protect this mark from being removed, while HDAC2 competes with ATAD2 for the target acetyl group to fine-tuning the balance of H4 acetylation 71. In addition, ATAD2 also has a very obvious response to DNA damage and repair (DDR). After DNA damage drug treatment or radiotherapy, ATAD2 can be highly expressed by ATM/ATR pathway. When activated, ATAD2 can accelerate CHK1/2-regulated DNA damage repair signaling and may promote *BRCA1* expression by increasing the recruitment of H3K4 methyltransferase MLL1 at the *BRCA1* promoter, which may further promote the homologous recombination repair (HRR) of the damaged DNA 15. For instance, in pancreatic cancer, ATAD2 inhibition increases the sensitivity of pancreatic cancer cells to **gemcitabine** and radiotherapy. When ATAD2 is inhibited, the DNA damage of the cancer cells is significantly intensified, while the expression of DNA damage repair proteins is significantly down-regulated, further demonstrating the crucial role of ATAD2 in DDR 72. Thus, due to its important role in DNA replication and DDR, ATAD2 may be a potential candidate target in cancers that are susceptible to DNA damage.

#### 4.2.3 ATAD2 in Cell Cycle Regulation

Strikingly, abnormal cell cycle is also a major feature of cancer, and more and more evidence also shows that ATAD2 plays an important role in the regulation of various stages of cancer cell cycle. In CC and GC, ATAD2 was significantly over-expressed. After ATAD2 knockout, the cancer cells showed significant G1/S cell cycle arrest, which was related to the Rb-regulated cell cycle pathway 40, 73. Rb signaling is a key signaling point in cell cycle regulation, which is mainly composed of CDKN, D-type cyclins, cyclin-dependent protein kinases (CDK4, CDK6), the Rb pocket protein family (Rb, p107, p130), and the E2F transcription factor family (E2F1-8 and DP1-2). Of them, Cyclin D and CDK4/6 disrupt the Rb-E2F interaction through phosphorylation of Rb proteins, resulting in activation of E2F-regulated gene expression 74. Cyclin D1 and CDKs can drive G1 to S phase progression by phosphorylating Rb 43. Interestingly, cyclin D1, E2F1 and Cyclin E proteins all showed a down-regulation trend after ATAD2 knocking out, which further indicated that ATAD2 could regulate cell cycle progression by regulating the expression of key proteins of Rb-E2F1 signaling pathway 40. Notably, ATAD2 can also regulate transcription factors such as E2F and MYC to control the cell cycle 40. As to E2F, ATAD2 can interact directly with E2F and bind to H3K14ac via its bromine domain. During the transition from G1 to S, the interaction between ATAD2 and E2F reaches its maximum intensity. In this process, Host cytokine 1 (HCF-1)-MLL histone methyltransferase complex and nuclear hormone receptor coactivator ACTR also contribute to the recruitment of E2F targets by ATAD2 and the assembly of chromosomes 9. And as to MYC, ATAD2 cooperates with MYC in activating transcription, and it is essential to the efficient transcriptional activation of numerous MYC target genes 64, including key cell cycle regulators, such as CDKs, cyclins, and E2F transcription factors 75. In addition, B-MYB is a member of the Myb family of highly conserved transcription factors, which not only regulate the cell cycle but are also ubiquitously expressed in strong proliferative cancer cells. High expression of ATAD2 can facilitate the up-regulation of B-MYB 57, while ATAD2 silencing significantly inhibits the expression of B-MYB and some cell cycle proteins, such as cyclin E1, cyclin B1 and cyclin A2 in malignant tumors 76. Accordingly, ATAD2 can encourage the process of cell cycle through a variety of aspects to promote cancer progression.

#### 4.2.4 ATAD2 in Gonadal Hormone Signaling

As we all know, gonadal hormones are essential for human growth and development. In recent years, more and more studies have illustrated that abnormal gonadal hormone signaling pathway is also an important risk factor for the onset and development of various gonadal hormone-related tumors, especially breast cancer and prostate cancer 77, 78. Excitingly, ATAD2 has also been identified to be involved in the regulation of gonadal hormone signaling in cancer progression.

##### Estrogen Receptor

ATAD2 is a transcriptional co-regulator of ER, which can affect tumorigenesis by enhancing the transcription of ER target genes 7, 9. Upon estrogen stimulation, ATAD2 is selectively recruited to the promoters of ERα target genes, such as cyclin D1, c-Myc and E2F1, which are required for estrogen-induced cancer cell proliferation. Intriguingly, ATAD2 does not occupy the promoters in ERα target genes but promotes the recruitment or assembly of the ERα-CBP complex at the chromatin, which is crucial for histone modification 7. In addition, ATAD2 participates in regulating the E2-dependent recruitment of E2F and MLL1 at the promoters of the kinesin genes (such as Kif4A, Kif15, Kif20A, and Kif23), promoting their expression. Of note, the promotion of kinesin expression mediated by ATAD2-E2F1-MLL1 is required for BC cell proliferation and survival and is associated with the prognosis of BC patients 12. Besides, ATAD2 can control the photo-oncogene ACTR/A1B1 expression by recruiting to its promoter 79, while ACTR can interact with estrogen-bound ER to strongly co-activate the transcription of ER target genes 80. Additionally, ACTR can directly interact with E2F1 and then locates in the promoter of E2F target genes to promote their expression 81. It is worth noting that the ACTR-E2F pathway can promote the proliferation of BC even without the stimulation of estrogen and the participation of ER 81. Therefore, the ATAD2-ACTR-E2F pathway may still have potential targeting value in the treatment of ER-negative BC.

##### Androgen Receptor

Similar to ER, androgen receptor (AR) is mainly activated by the androgen steroid hormones testosterone and 5α-dihydrotestosterone (5α-DHT), which is the major factor promoting the proliferation of prostate cancer 82. Transcriptional activation or repression mediated by AR, as a member of the nuclear hormone receptor superfamily, requires several co-regulators, including ATAD2 83. It has been found that inhibition of ATAD2 strongly impedes the proliferation of prostate cancer cells and leads to apoptosis enhancement. In addition, overexpression of ATAD2 enhances the expression of a multitude of AR target genes, such as *IGF1R, IRS-2, SGK1, E2F1, Cyclin A2, Cyclin D3,* and *Survivin*. Mechanistically, ATAD2 directly interacts with the DBD-hinge region of AR via its NH2 terminus to facilitate its transcriptional activity and enhance androgen-stimulated gene expression 83. Likewise, ATAD2 can be also regulated by the E2F1 transcription factor, and E2F1 interacts with AR on the ATAD2 promoter through a chromatin loop to activate transcription upon androgen stimulation 65. Interestingly, in the case of androgen deficiency, KDM8 could interact with AR to enhance the expression of androgen-responsive genes, such as ATAD2 and EZH2. Impressively, EZH2 can also be activated by AR via ATAD2 binding to its promoter 19. Therefore, ATAD2 can assist the expression of androgen receptor target genes from multiple paths.

Together, ATAD2 acts as a transcriptional co-activator of ER and AR, regulating the expression of ER/AR-targeted genes to control cancer cell proliferation and survival 84, even without gonadal hormone stimulation. Therefore, it is not difficult to speculate that in cancer with negative or positive gonadal hormone receptors, ATAD2 may both have strong potential targeting value.

#### 4.2.5 ATAD2 in MAPKs and PI3K/AKT/mTOR Pathways

As well-known cancer-related family proteins, MAPK cascades and AKT signaling play important roles in a variety of cellular processes. Notably, MAPK and PI3K/AKT/mammalian target of rapamycin (mTOR) signaling have a close relationship, and the complex network they constitute is of great significance in cancer cell proliferation, angiogenesis, metastasis, metabolic changes, and response to anticancer drugs 85-87. Excitingly, ATAD2 is also involved in regulating this complex network that governs the fate of cancer. In ovarian cancer, ATAD2 deletion attenuates the MAPK pathways by decreasing the phosphorylation levels of JNK1/2 and ERK1/2 MAPK 5. Likewise, ATAD2 inhibition by miR-200b impedes the protein expression levels of PI3K and p-Akt, thereby inhibiting the proliferation and apoptosis of ovarian cancer cells. Conversely, highly expressed ATAD2 protein can promote the protein expression levels of PI3K and p-Akt to promote ovarian cancer progression 50. In HCC, ATAD2 deletion could activate p38 MAPK to activate apoptosis. Mechanistically, ATAD2 directly interacts with MKK3/6, which is the upstream regulator of p38 MAPK 88. Similarly, ATAD2 can also regulate the proliferation, tumorigenicity, and migration of cancer cells through PI3K/AKT pathway in lung adenocarcinoma 39, 53. In this process, ATAD2 significantly positively upregulates the expression of p-AKT, GLUT1, and HK2, which also contribute to glucose uptake and metabolism 39. In addition, ATAD2 inhibition by miR-506 can improve lung adenocarcinoma's sensitivity to cisplatin-based hyperthermia by inhibiting the ATAD2-PI3K-AKT pathway 53. Encouragingly, a recent study found that AM879, an inhibitor of ATAD2, blocks the PI3K-AKT-mTOR pathway through ATAD2 inhibition, thereby facilitating breast cancer cell apoptosis and autophagy-associated cell death 89. Unfortunately, but still hopeful, although the involvement of ATAD2 in the regulation of MAPKs and AKT signaling pathways is well established, the exact mechanisms are still in their infancy.

#### 4.2.6 ATAD2 in HIF1α-mediated Hypoxia Signaling and EMT Pathways

In cancer, another major issue that cannot be ignored is cancer microenvironment and cancer metastasis 90, 91. Of note, the hypoxic microenvironment is necessary for the development of solid tumors and contributes to tumor EMT, angiogenesis, and metastasis 92. EMT is a biological process in which epithelial cells are transformed into cells with a mesenchymal phenotype by a specific procedure. In epithelial-derived malignancies, the migration and invasion abilities of tumor cells are closely related to EMT 93. Intriguingly, ATAD2 has also been found to contribute significantly to cancer response to hypoxia and EMT. In stomach cancer and lung cancer, ATAD2 and HIF-1 α always highly expressed 59, 94. Mechanistically, ATAD2 is a hypoxia-responsive gene, and its promoter contains an HIF1α binding site (HBS) and HIF1α ancillary site. Under hypoxic conditions, HIF1α binds to the ATAD2 promoter, and the expression of ATAD2 is encouraged, which further promotes the cancer proliferation and migration process 59. In lung cancer, HIF-1α activates ATAD2 to enhance cancer development, which may further facilitate mitochondrial ROS production that contributes to enhancing cancer cell stemness 94. As to EMT, the most striking sign is the upregulation of N-cadherin and Vimentin protein expression and downregulation of E-cadherin 43. Notably, inhibition of ATAD2 can inhibit EMT by reducing the expression of EMT regulators such as slug and snail in OSCC 46. In addition, ATAD2 silencing inhibits EMT through upregulation of E-cadherin and downregulation of vimentin in CRC cells, thereby preventing tumor invasion and migration 38. Besides, the activation of PI3K-AKT-mTOR and MAPKs signaling by ATAD2 may contribute to EMT. Interestingly, the TGF-β1 cascade activation by ATAD2 may also contribute to the EMT process 41. It is worth noting that the underlying mechanism of how ATAD2 regulates these key EMT regulators have not yet been fully revealed (TGF-β1-SMAD3 may contribute a lot), but ATAD2 does contribute significantly to cancer progression by regulating HIF1α-mediated hypoxia signaling pathways and EMT pathways.

#### 4.2.7 ATAD2 in Other Cancer-associated Signaling Pathways

Despite the research history of ATAD2 is not long, with the deepening of research, ATAD2 has also been found to be involved in many other cancer related pathways, including hedgehog (HH), TGF-β1, YAP/Hippo, p53 and NF-κB signaling pathways.

HH signaling pathway is a highly conserved evolutionary pathway that involves various stages of carcinogenesis 95. Interestingly, ATAD2 can regulate the HH pathway to promote cancer development, angiogenesis, invasion, and metastasis. For instance, silencing ATAD2 can impede the HH signaling pathway by reducing the expression of Gli1, SMO, and PTCH1 48. Additionally, ATAD2 and MYC can co-regulate the expression of the HH pathway-related genes *SMO* and *Gli1* through MYC-interacting zinc finger protein 1 (MIZ1) and accelerate the activation of the Hedgehog pathway 96. For example, miR-186 can inhibit HH pathway by impairing the expression of ATAD2, which is beneficial to the inhibition of angiogenesis of retinoblastoma 48. Similar to the HH pathway, TGF-β1 signaling is also an important developmental signaling pathway that controls cell fate 97. While ATAD2 directly interacts with C/EBPβ, the transcriptional activator of TGF-β1, to promote its nuclear translocation and directly bind to the promoter of TGF-β1 to activate TGF-β1 expression. Then, the activation of ATAD2-TGF-β-SMAD3 signaling may further enhance the EMT of ESCC 41. YAP/Hippo signaling is another key pathway that controls organ development, its incontrollable is closely related to the occurrence and development of cancer 98. Recently, ATAD2 was found to be directly regulated by YAP1, which contributes to the tumorigenesis of head and neck squamous cell carcinomas. During this process, YAP, TAZ, and TEAD form transcription complexes and recruit BRD4 to promote the open state of chromatin and promote the transcriptional activation of ATAD2 63. Furthermore, p53, as the most well-known tumor suppressor protein, its important role in cancer that needs not to be explained. Importantly, ATAD2 has also been reported to be involved in the p53-regulated apoptotic cascade. In the presence of wild-type p53, silencing of ATAD2 induces the phosphorylation activation of p53 in HCC cells, as well as the activation of a variety of pro-apoptotic proteins, including Puma, Bax, and so on 88. Unfortunately, the exact regulatory mechanisms of ATAD2 on p53 remain to be fully elucidated. Besides p53, NF-κB signaling pathway also participates in the regulation of apoptosis. Meanwhile, it is also known for its role in the cellular response to inflammation and immunity 99. Hopefully, ATAD2 may regulate NF-κB signaling through its target protein histone methyltransferase NSD2. As a co-activator of NF-κB, NSD2 directly binds to NF-κB and is recruited to the NF-κB target gene promoter. In addition, co-activator p300 is also involved in this process, which further activates the NF-κB signaling 100.

Hence, the relevant signal network with ATAD2 as the core plays an important role in various pathways that cancer depends on for survival. Undoubtedly, ATAD2 has an important hopeful targeting value in cancer therapy, and these biological processes regulated by ATAD2 are the molecular basis for ATAD2 to become a promising candidate drug target in cancer therapy.

## 5. Targeting ATAD2 with pharmacological small-molecule compounds in cancer therapy

Given the comprehensive cancer-related functions of ATAD2, disrupting the ATAD2-acetyl-lysine interactions has emerged as a potential therapeutic target in cancer, and a lot of pioneering efforts have been made to develop ATAD2 inhibitors from both pharmaceutical and academic settings.

### 5.1 The discovery of fragment hits for ATAD2 Bromodomain

The discovery of hits with potential activity is crucial for the development of targeted drugs. In 2014, Apirat Chaikuad *et al*. first discovered nine novel hits with weak affinity for ATAD2 by crystallographic fragment screening, including thymidine (**1**) and 3-methylquinolin-2(1H)-one (**2**) with K_d_ value of 10 mM and 0.6 mM respectively 101. The crystal structures of fragments showed the endyllamide group of 3-methylquinolin-2(1H)-one (**2**) initiated two conservative hydrogen bonds with the ASN1064 residue which is the crucial active center to adopt the acetyl-lysine of substrates. Furthermore, the methyl group in the adjacent position occupied a tight hydrophobic space, which enhanced the affinity to ATAD2. 3-methylquinolin-2(1H)-one (**2**) is a novel acetyl-lysine mimetic ligand, offering a promising chemical starting point for the development of more potent ATAD2 inhibitors. Another fragment-based approach was performed to discovery new ATAD2 fragment hits, the results revealed three hits (Compound **3**, **4**, **5**) were found by NMR spectroscopy. According to the crystal structure of **3** with ATAD2, a conserved hydrogen interaction was formed between the carbonyl group and ASN1064 residue and the methyl group played an important role in the simulation of the acetyl group 102. To screen new ATAD2 inhibitors, structure-guided studies were conducted by John E. Ladbury from the MD Anderson Cancer Center. They elucidated an unexpected conformational change of the conserved ASN1064 residue plays an important role in driving the peptide-binding conformation remodeling. The methyltriazolium- and dimethylisoxazole-containing ligands (**6**, **7**) were identified as potential ATAD2 inhibitors by imitating acetyl group 103. The conformational selection of the ATAD2 bromodomain was explored through the synergistic combination of molecular dynamics and protein crystallography. The gatekeepers Ile1074, Val1008, and Val1013 residues played an essential role in assuming multiple orientations for different ligands. It is a novel strategy to discover potent and selective ATAD2 inhibitors by coupling the ZA-loop rigidification and the rearrangement of the hydrophobic side chains. Based on the strategy, a potent ATAD2 inhibitor (**8**) was screened with a K_d_ value of 15 μM 104. The linker of acid amides formed two hydrogen bonds with ASN1064, and the piperazine group initiated a hydrogen interaction with ASP1071 located at the BC loop, which provided a promising scaffold to further optimize selective ATAD2 inhibitors. Based on the resolution of binding modes of ligands and ATAD2 bromodomain, they adopted a structure-based virtual screening strategy to discover a novel ATAD2 inhibitor (**9**, **AM879**) with an IC_50_ value of 3565 nM. **AM879** significantly inhibited breast cancer cell proliferation with an IC_50_ value of 6.0 μM and induced significant apoptosis and autophagy via PI3K-AKT-mTOR signaling in MDA-MB-231 cells 89. Collectively, although these fragment hits and leads did not show a very strong affinity for the ATAD2 bromodomain, they promoted the understanding of the ligand and receptor binding modes, which lays a key foundation for the discovery of highly selective novel targeting ATAD2 inhibitors.

### 5.2 Azepines derivatives

ATAD2, as an epigenetic bromodomain-containing oncology target overexpressed in many cancers, has attracted widespread attention among pharmaceutical companies. The researchers from AstraZeneca conducted a high throughput screen by TR-FRET assay, a tetrahydroisoquinoline derivative (**10**) with IC_50_ value of 21.2 μM was screened from the AstraZeneca screening collection including 1.8 M samples 29. The X-ray structure showed **6** occupied in the acetyl-lysine binding site of ATAD2, two key hydrogen interactions were observed between the phenol and Asn1064, and two additional hydrogen interactions were formed between the tetrahydroisoquinoline group and GLU1017, ASP1014 from ZA loop associated with the selectivity for ATAD2. To improve the affinity for active Asn1064, the phenol group of **10** was replaced by a pyridone warhead inspired by the previous fragment hit (**2**). Derivative **11** presented a significantly improved potency with an IC_50_ value of 11.8 μM. More pyridine derivatives were designed and synthesized, such as pyrrolopyridinone derivative (**12**, IC_50_ value of 1.48 μM). Compound **13** showed the most potent against ATAD2 in this subseries with an IC_50_ value of 0.078 μM, which combines a 2R-methyl with an OCF_3_ substituent on the pyridoindolone ring. As expected, the carbonyl of pyridone formed a hydrogen bond with Asn1064 and the methyl group occupied the hydrophobic pocket. Furthermore, the phenol group of 10 was replaced by triazolopyridazine, a classical acetyl group mimics, producing a new series of TAD2 inhibitors. 3-methyl-[1,2,4]triazolo[4,3-b]pyridazin-8-amine derivative (**14**) played a significantly more potent affinity against ATAD2 than its pyrrolopyridinone analog **11**. The triazolopyridazine group initiated two conserved hydrogen bonds with ASN1064 and additional two hydrogen bonds were preserved between tetrahydroisoquinoline group and GLU1017, ASP1014 residues. Next, 1-methylpiperidin-4-amine moiety was introduced to product **15** with a similar potency as **14**. The 2-fluoro-2-methyl-propyl-piperidine derivative (**16**, **AZ13824347**) showed the most potent activity with an IC_50_ of 7 nM, and antiproliferatory activity with a pIC_50_ of 6.9. Due to the good potency, selectivity, and permeability of **AZ13824347**, AstraZeneca is conducting a comprehensive pharmaceutical study on **AZ13824347**, expected to enter into clinical trials.

### 5.3 3-methylquinolinone derivatives

The 3-methylquinolin-2(1H)-one (**2**) was an important fragment hit to optimize potent selective ATAD2 inhibitors which caused the extensive interest of GlaxoSmithKline researchers. Firstly, N,N-dimethylethane-1,2-diamine group was incorporated into the 8th site of quinolinone core to yield **17** with pIC_50_ of 3.3 μM. The endyllamide group formed two conserved hydrogen bonds with ASN1064, and the NH moiety also initiated a hydrogen interaction with ASN1064 enhancing the affinity. The methyl group occupied the hydrophobic pocket and the N, N-dimethylethane-1,2-diamine side chain interacts with ASP1071 by a hydrogen bond. The side chain was replaced with piperidin-4-amine group to product **18** which presents an improved affinity with a pIC_50_ of 4.9 μM. To optimize the selectivity for ATAD2, 3-methoxypyridine group was incorporated into the 5th of 3-methylquinolinone to form a hydrogen bond with ASP1014 located in the ZA loop, **19** presented a significant affinity for ATAD2 with pIC_50_ of 1.3 μM 105. Based on the analysis of **18** and ATAD2, a potential hydrophobic pocket closing to the RVF shelf of the ZA loop was found. A cyclic sulfone group was incorporated into the 3'-site of piperidine to yield **20** with pIC_50_ of 5.6 μM, initiating additional three hydrogen bonds with ARG1077 and ARG1007. Based upon the structure of **19**, 3-methylpyridine group was also induced the 5th of 3-methylquinolinone, **21** showed the most potent affinity with IC_50_ of 130 nM and 100-fold BET selectivity 106. On this basis of **21**, the cyclic sulfone group was replaced by the CF_2_ group to yield **23 (GSK8814)** which was identified as the first reported low-nanomolar, selective, and cell-permeable chemical probe for ATAD2 Bromodomain 107. To make further efforts, the researchers hypothesized that constrained bridged piperidine derivatives would show greater selectivity over the BET bromodomains. The piperidine was replaced with tropane yield **22** which is a potent ATAD2 inhibitor with IC_50_ of 100 nM and has fairly good BRD4 selectivity 25.

### 5.4. Imidazolidine-2,4-dione and furane derivatives

In addition to the acetyl-mimetic agent, imidazolidine-2,4-dione and furane derivatives were also developed as potent ATAD2 inhibitors with atypical binding modes. The researchers from AstraZeneca conducted a high throughput screen by TR-FRET assay, NMR, and SPR, 16 hits were obtained from the 1.7 million collections 108. An imidazolidine-2,4-dione derivative (**24**) presented the most potent affinity for ATAD2 with pIC_50_ of 5.9 μM. The Br substituted (**26**) displayed an improved affinity with pIC_50_ of 6.5 μM. Further, the 5-cyclopropyl-5-methylimidazolidine-2,4-dione group was replaced with imidazolidine-2,4-dione to yield **25**. Unfortunately, the activity of **25** showed no significant improvement. According to the X-ray structure of **25** and ATAD2, the sulfonyl group initiated a hydrogen bond with TYR1021, and three hydrogen interactions were observed between imidazolidine-2,4-dione group and ARG1007, LYS1011 residues. No interaction was observed between the ligand and ASN1064, which is a significant difference from the common ATAD2 inhibitors. These findings suggested that imidazolidine-2,4-dione derivatives bind to ATAD2 with a novel binding mode. Additional two derivatives were designed and synthesized 109, **27**, an (S)-3-methylpyrrolidine substituted, presented an improved activity with pIC_50_ of 7.2 μM. The sulfonyl group initiated a hydrogen bond with TYR1021, and four hydrogen interactions were observed between the side chain and residues GLU1017, ASP1014, LYS1011, and ARG1007. The 5-cyclopropyl-5-methylimidazolidine-2,4-dione group was substituted with 5-isopropyl-4,5,6,7-tetrahydro-[1,2,3] triazolo[4,5-c]pyridine to yield **28**, and **28** showed the most potent affinity for ATAD2 with pIC_50_ of 7.5 μM.

Pharmaceutical giant Bayer has also made significant efforts to develop novel ATAD2 inhibitors as innovative anti-tumor drugs. A series of furane derivatives were discovered by a DNA-encoded library screen, **29** displayed an IC_50_ value of 1.89 μM against ATAD2. A shorter formylamine derivative (**30**) presented slightly increased inhibitory activity of ATAD2 with an IC_50_ value of 0.74 μM. A 2-(piperidin-4-yl)ethan-1-amine moiety was incorporated to product **31**, displaying a potent inhibitory potency of ATAD2 with an IC_50_ value of 0.086 μM 110. The cyclohexane-1,4-diamine substituted (**32**, **BAY-850**) showed the most potent inhibitory activity with an IC_50_ value of 0.02 μM. **BAY-850** is widely used as an isoform-selective ATAD2 chemical probe to explore the biological function of ATAD2 because of its potent activity against ATAD2 24. However, the binding mode of **BAY-850** and ATAD2 has not yet been resolved, some researchers put forward the hypothesis that **BAY-850 could** specifically induce ATAD2 bromodomain dimerization and prevented interactions with acetylated histones, but more solid evidence needs to provide.

### 5.2 AAA + ATPase domain

As a candidate drug target, in addition to the abovementioned BRD region, ATAD2 also exists as an important domain responsible for ATP binding, hydrolysis, and polymerization, ---AAA + ATPase domain. The importance of AAA + ATPase domain for estrogen-stimulated-mediated gene expression, as well as efficient binding to chromatin, has been demonstrated by specific mutations. For instance, studies have shown that mutations in the AAA + ATPase domain, which is responsible for mediating the multimerization of ATAD2, affect the recruitment and/or occupancy of CBP at the promoter of Erα 7. Despite the biological functional importance of the AAA + ATPase domain, small molecules developed to target it still need to be further explored, possibly because the spatial structure of the AAA + ATPase domain of ATAD2 has not been resolved and because of the high homology with other AAA + ATPase family members. To date, small-molecule compounds targeting AAA + ATPase family members are gradually being reported. Dynein, an AAA + ATPase, is responsible for powering all microtubule-negative-oriented movements within eukaryotic cells, which plays a crucial role in a variety of cellular functions 111. In a study of Caenorhabditis elegans, deficiency of either light or heavy chains of dynein at the nuclear envelope inhibited physiological apoptosis in phagocytic defective mutants, involving the CED-4/Apaf1 protein 112. A novel aminothiazol inhibitor of cytoplasmic dynein 1/2, Dynarrestin, blocks smooth-mediated Hedgehog signaling by transmembrane receptors 113. Furthermore, another AAA + ATPase family member, p97, has been greatly developed in recent years. Accumulating studies have found that the expression of p97 is upregulated in a variety of cancers and is closely related to the poor prognosis of diseases such as gastric cancer and liver cancer 114-116. NMS-873, a potent allosteric inhibitor of p97, activates unfolded protein responses, interferes with autophagy and promotes cell death in CRC HCT-116 cells 117.

In short, the AAA + ATPase domain still has a long way to go as an alternative strategy for small-molecule compounds to target ATAD2. It is hoped that the abovementioned successful examples of developing targeted dynein and p97 will provide new inspiration for the design of compounds targeting ATAD2.

## 6. Conclusion and perspectives

Up to present, the involvement of ATAD2 in epigenetic modifications that promote cancer cell proliferation and its clinical significance in diagnosis and prognosis has been continuously revealed, making it an emerging and promising cancer therapeutic target. For instance, ATAD2 is highly expressed in breast cancer, and its expression level is remarkably correlated with tumor grade 22. In addition, inhibition of ATAD2 expression can effectively inhibit the proliferation of prostate cancer cells and induce apoptosis 27. To further evaluate the potential of ATAD2 as an anticancer target, it is essential to understand its biological role in cancer. In this thesis, we describe in detail the structure of ATAD2, which is mainly composed of BRD and AAA + ATPase domains, and its important biological roles in cancer, including participating in epigenetic modifications and regulating downstream oncogene expression and signaling pathways. Notably, ATAD2 functions as a coactivator of epigenetic readers or transcription factors in the Rb/E2F-CMYc pathway, PI3K/AKT/mTOR, and other signaling pathways. Finally, we summarized small-molecule inhibitors targeting BRD in ATAD2, such as GSK8814, AZ13824374, and BAY-850. Taken together, elaborating on the abovementioned contents will not only help to deepen the understanding of ATAD2 in cancer development but also provide an unprecedented opportunity for the development of anticancer drugs with new targets.

Anticancer therapy targeting ATAD2 is not a simple subject; thus far, there are still many problems and challenges to be solved. One of the best problems is the design of potent novel small molecule inhibitors targeting ATAD2. The BRD domain in ATAD2 is a promising target. Nevertheless, unlike the classical BRD4 domain, the BRD in ATAD2 replaces the WPF (Trp-Pro-Phe) motif with the RVF motif (Arg-Val-Phe) which is more flexible and hydrophilic, resulting in more challenging to engage in productive interactions when designing ligands. Another distinctive Arg1077 in ATAD2 in place of BRD4 BD1 residue Met149, leading to a more hydrophilic binding site in ATAD2. Moreover, the ZA loop of ATAD2 is two residues shorter than that of BRD4, contributing further to the relative solvent exposure and hydrophilicity of its KAc site 103, 107, 118, 119. Similarly, the AAA + ATPase domain also faces challenges in drug discovery. For example, the lack of a detailed spatial crystal structure of this domain and its high similarity to other AAA + ATPase family members implies that a series of adverse reactions such as poor selectivity and side effects may occur. Second, the reported small-molecule inhibitors, such as GSK8814 and AM879, are still in their infancy, and the anticancer mechanism and pharmacodynamic studies should be further explored to pave a credible path for them to become effective anticancer drugs.

In conclusion, the development of targeted ATAD2 inhibitors is a daunting and creative task that will require the combined efforts of basic pharmacology, structural biology, and proteomics to usher in an unprecedented anticancer drug era. Increasing pharmaceutical giants, such as AstraZeneca, Bayer, and GlaxoSmithKline, have entered the development of ATAD2-targeted drugs, and the clinical application prospect of ATAD2-targeted small-molecule inhibitors is promising.

## Figures and Tables

**Fig 4:**
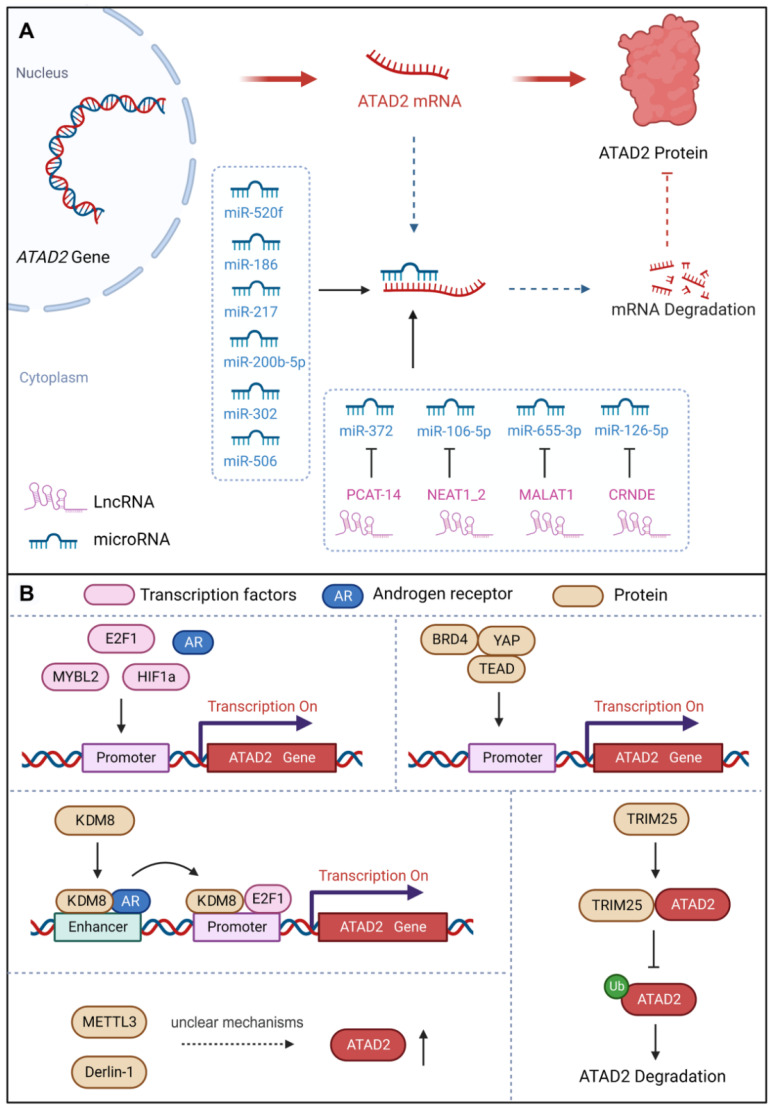
** Upstream regulators of ATAD2.** (A) Post-transcriptional regulation of ATAD2 expression via miRNAs and long non-coding RNAs. (B) Transcription factors, transcription co-activators, and some proteins can regulate ATAD2 expression or stability.

**Fig 5:**
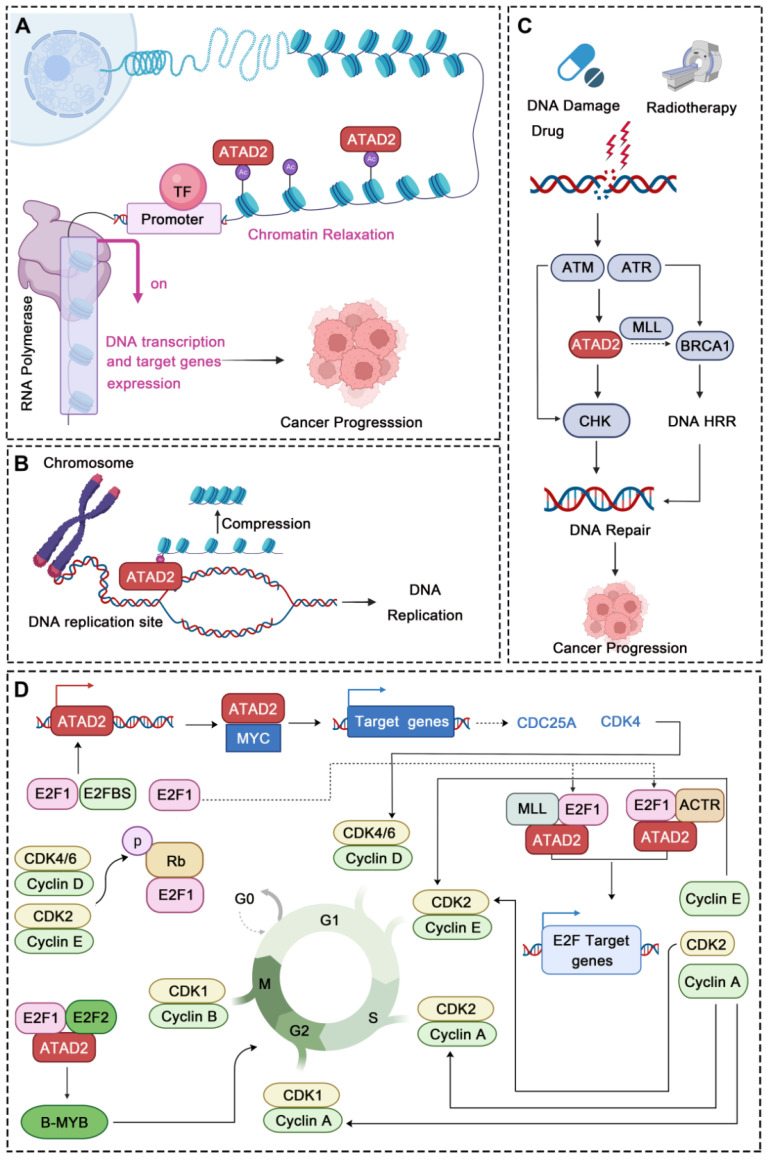
** ATAD2 regulates chromatin remodeling, DNA Replication, DNA Damage and Repair, and cell cycle.** (A) ATAD2 promotes chromatin remodeling by binding to the acetylation site of histones. This binding leads to chromatin relaxation and promotes DNA transcription and gene expression. (B) ATAD2 regulates DNA replication through interaction with the di-acetylation mark and binds to newly synthesized histones, which may assist in heterochromatin compression. (C) ATAD2 enhances the DDR response via activation of the CHK1/2 and BRCA1. (D) ATAD2 facilitates the cell cycle progression mainly through MYC, b-MYB, and E2F activation. In this process, the activation of CDKs and cyclins is also regulated by ATAD2.

**Fig 6:**
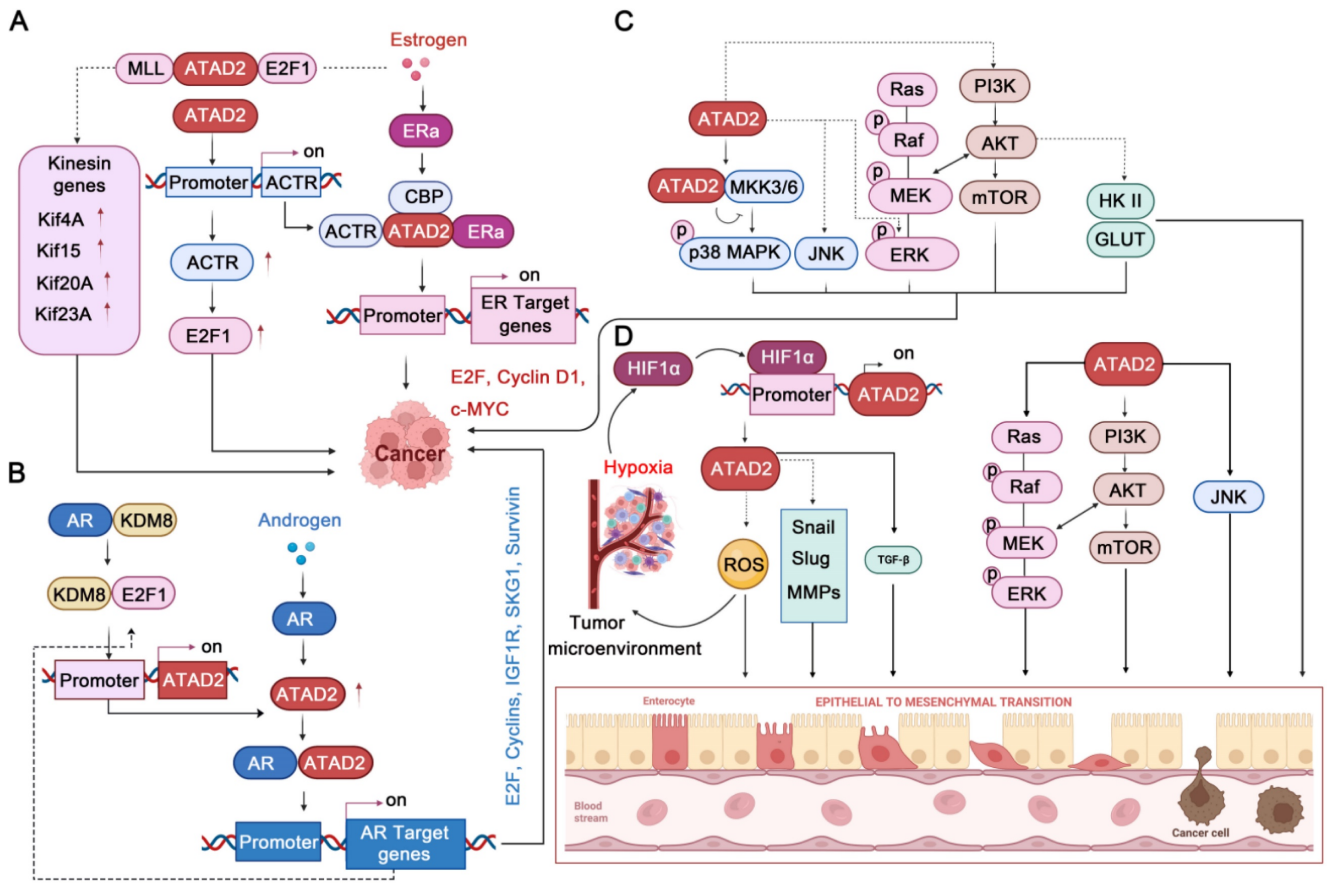
** ATAD2 in gonadal hormone MAPK, and PI3K/AKT/mTOR, HIF1α-mediated hypoxia, and EMT signaling pathways.** (A-B) ATAD2 plays an important role in gonadal hormone signaling by accelerating ER/AR target gene expression. (C) ATAD2 activates p38-MAPK, JNK1/2, and ERK1/2 MAPK to regulate cancer cell progression. In addition, ATAD2 activates PI3K-AKT-mTOR signaling to stimulate glucose metabolism and cancer progression. (D) HIF1α promotes ATAD2 expression to enhance EMT through up-regulating EMT-related genes, such as *Snail, Slug, MMPs.* Besides, ATAD2-regulated ROS production, ERK1/2, AKT and JNK1/2, and TGF-β pathways also contribute to enhancing EMT.

**Fig 7:**
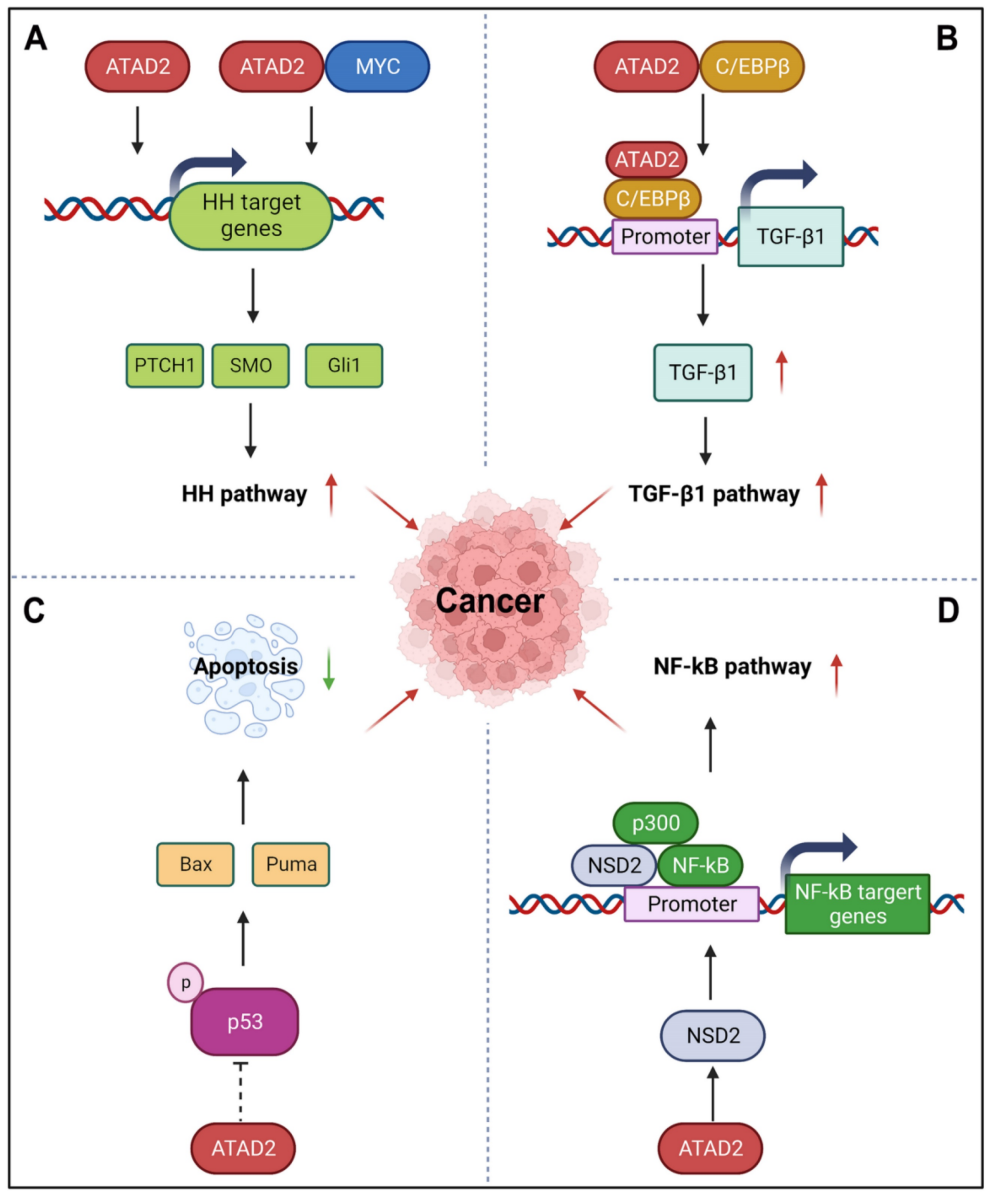
** ATAD2 in other signaling pathways in cancer.** (A) ATAD2 can stimulate or co-regulate the expression of some key HH pathway genes with MYC to activate the HH pathway. (B) ATAD2 promotes the expression of TGF-β1 via interacting with C/EBPβ, the transcriptional activator of TGF-β1, thus enhancing the TGF-β1 pathway. (C) ATAD2 inhibits the activation of p53 to suppress cancer cell apoptosis. (D) ATAD2 may activate the NF-κB signaling through the activation of NSD2, which is a co-activator of NF-κB.

**Fig 8:**
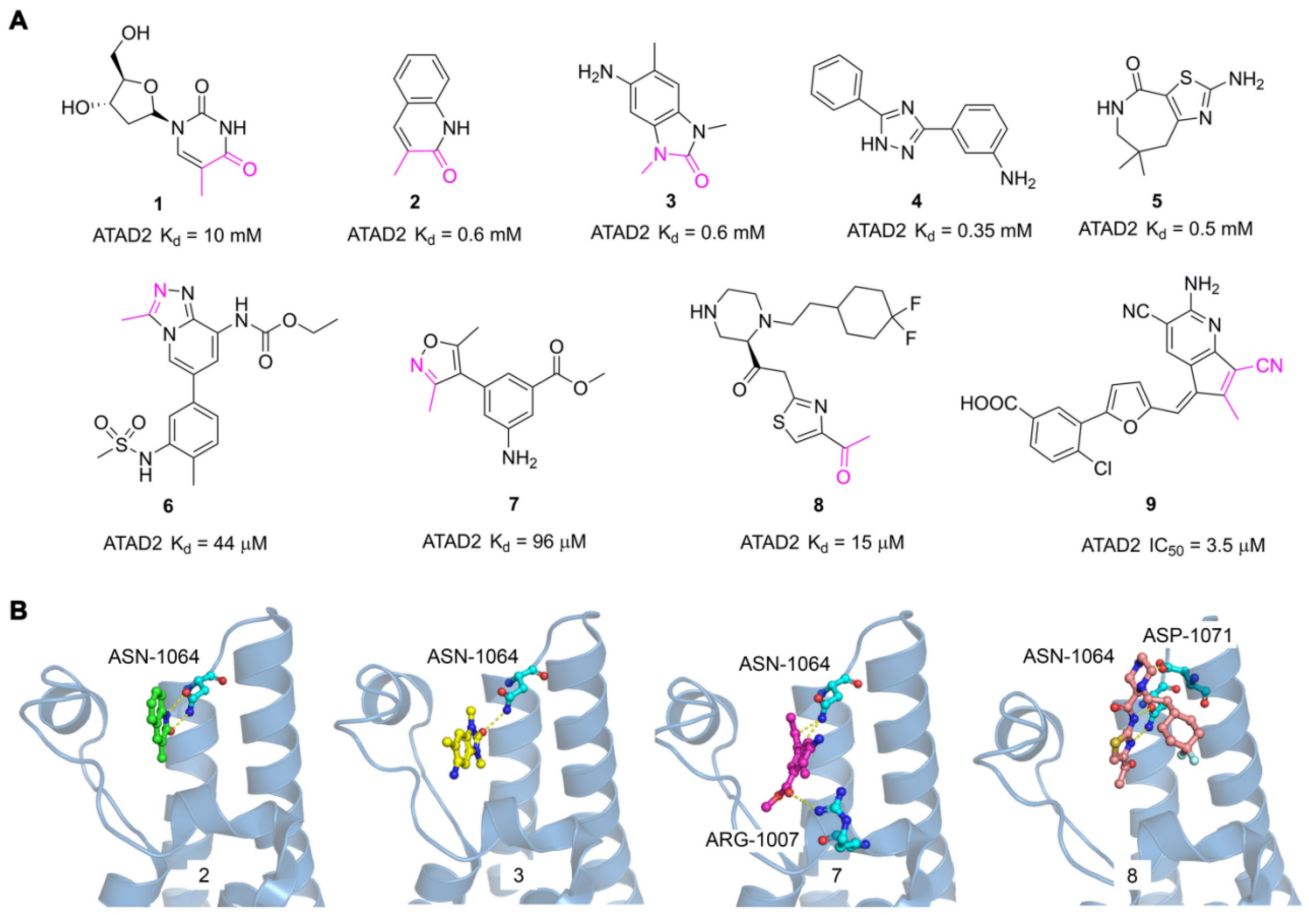
The reported fragment hits for ATAD2 Bromodomain and some representative X-ray structures of ligands and ATAD2 Bromodomain, including **2** (PDB code 5A5O), **3** (PDB code 4TYL), **7** (PDB code 4TTE), and **8** (PDB code 6HIE).

**Fig 9:**
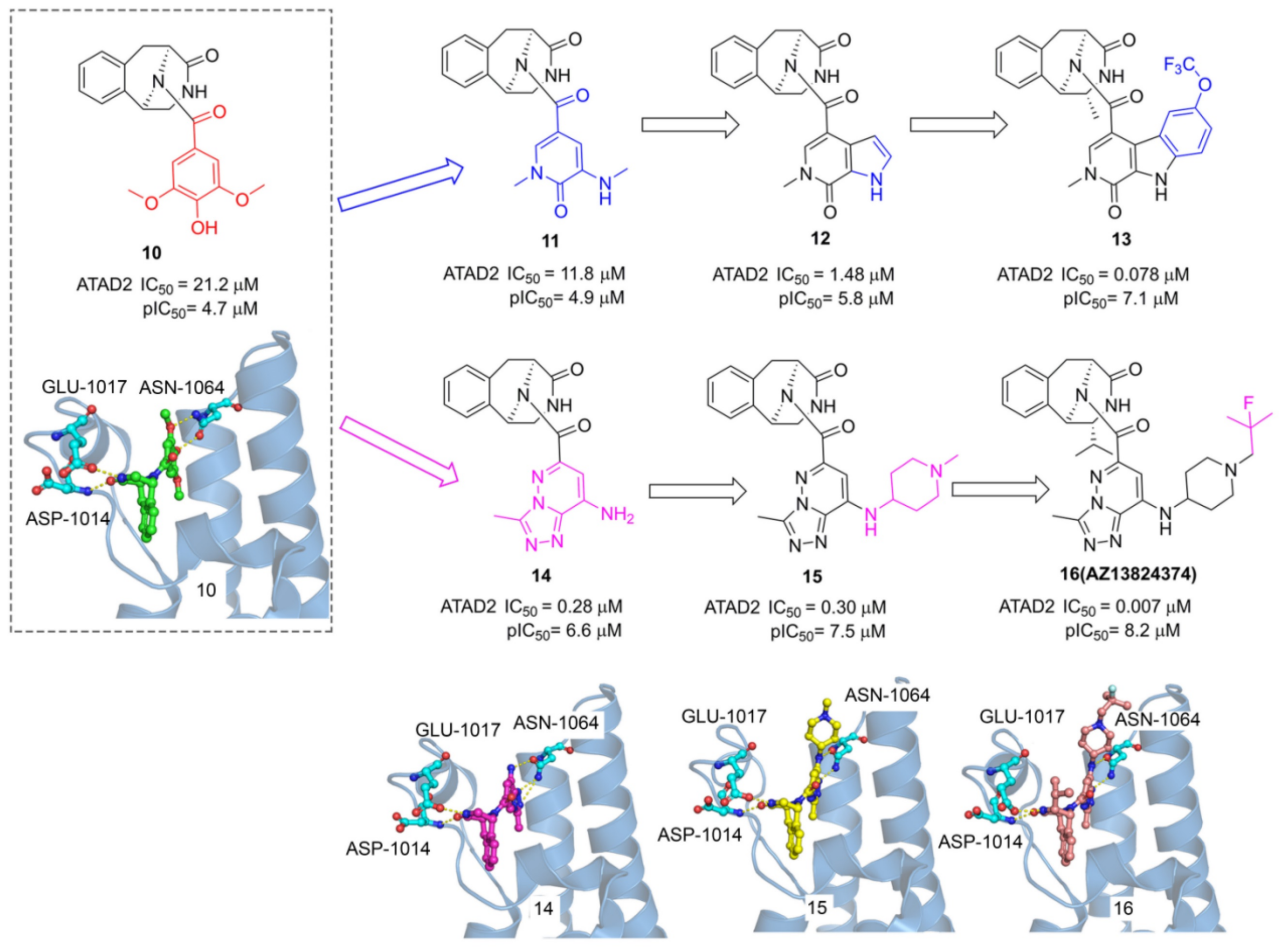
The chemistry structures of compounds** 10-16** and X-ray structures of ligands and ATAD2 Bromodomain, including **10** (PDB code 7Q6U), **14** (PDB code 7Q6V), **15** (PDB code 7Q6W), and **16** (PDB code 7Q6T).

**Fig 10:**
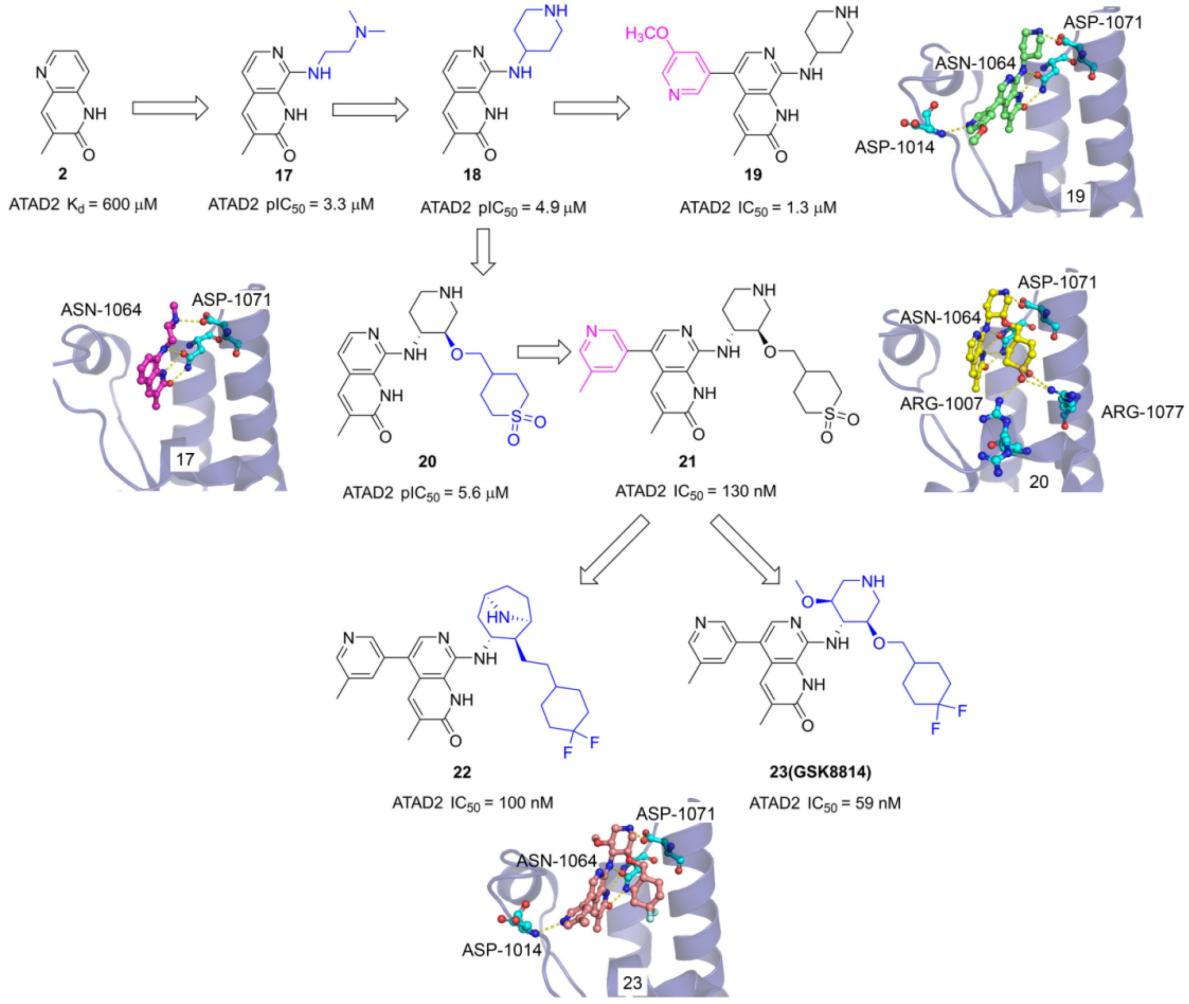
The chemistry structures of compounds** 17-23** and X-ray structures of ligands and ATAD2 Bromodomain, including **17** (PDB code 5A5P), **19** (PDB code 5A5R), **20** (PDB code 5A82), and **23** (PDB code 5lj0).

**Fig 11:**
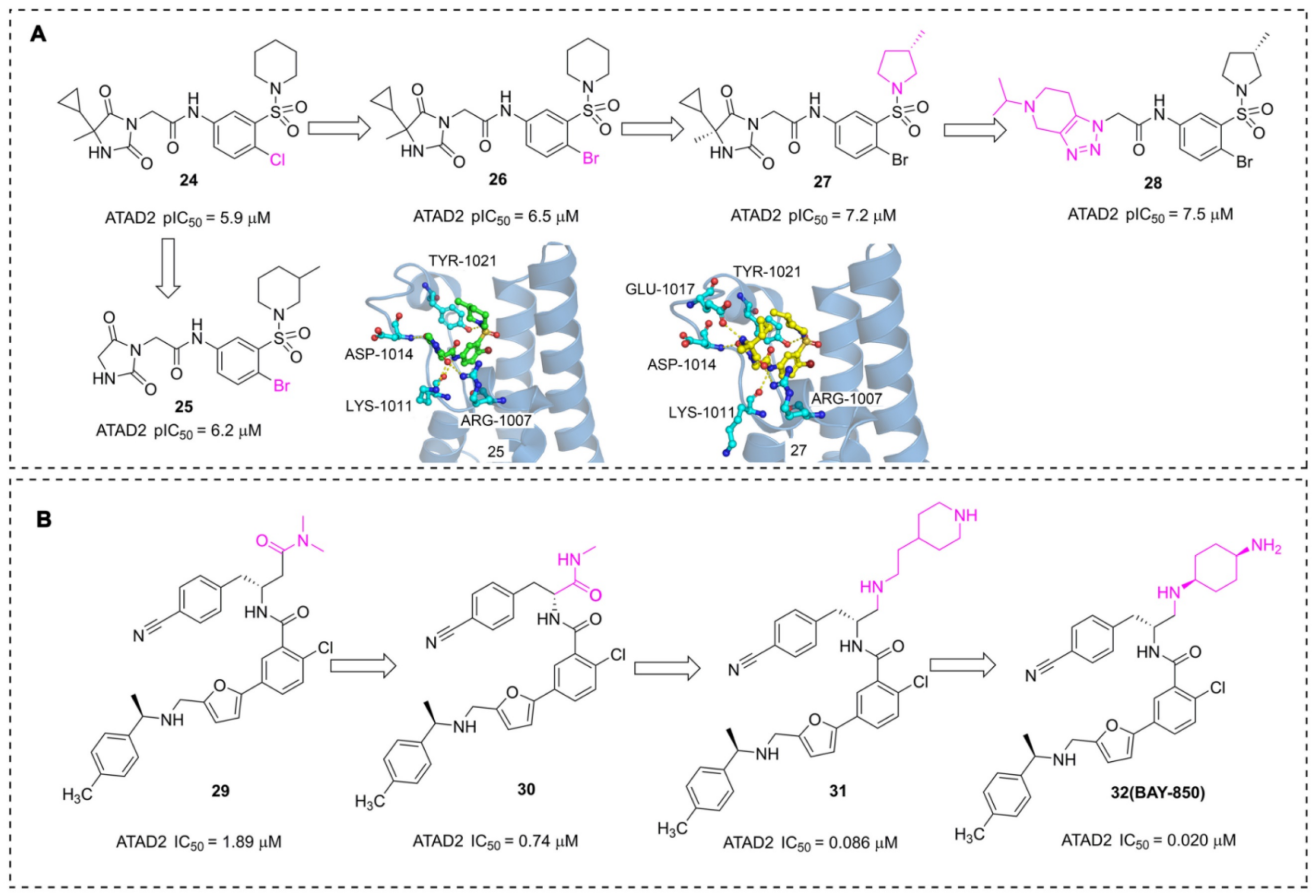
The chemistry structures of compounds** 24-32** and X-ray structures of ligands and ATAD2 Bromodomain, including **25** (PDB code 6s55), **27** (PDB code 6YB4).

**Table 1:** Upstream regulators of ATAD2 in cancer.

Name	Type	Positive/Negative	Mechanism	Cancer Type	Ref.
miR-520f	miRNA	-	miR-520f directly targets ATAD2 and downregulates its expression	Gastric carcinoma	49
miR-200b-5p	miRNA	-	miR-200b-5p directly targets ATAD2 and downregulates its expression	Ovarian cancer	50
miR-372	miRNA	-	miR-372 targets ATAD2 and suppresses its expression	Hepatocellular carcinoma	54
miR-186	miRNA	-	miR-186 directly targets ATAD2 and downregulates its expression	Retinoblastoma	48
miR-302	miRNA	-	miR-302 directly targets ATAD2 and downregulates its expression	Ovarian carcinoma	44
miR-217	miRNA	-	miR-217 targets ATAD2 and suppresses its expression	Pancreatic cancer	52
miR-506	miRNA	-	miR-506 could target and down-regulate ATAD2	Lung adenocarcinoma	53
PCAT-14	lnc RNA	+	PACT-14 upregulates ATAD2 expression by sponging miR-372	Hepatocellular carcinoma	54
NEAT1_2	lnc RNA	+	NEAT1_2 upregulates ATAD2 expression by sponging miR-106b-5p	Papillary thyroid cancer	55
miR-106b-5p	miRNA	-	miR-106b-5p directly targets ATAD2 and downregulates its expression	Papillary thyroid cancer	55
MALAT1	lnc RNA	+	LncRNA MALAT1 promoted ATAD2 expression via miR-655-3p	Retinoblastoma	43
miR-655-3p	miRNA	-	miR-655-3p directly targets ATAD2 and downregulates its expression	Retinoblastoma	43
CRNDE	lnc RNA	+	CRNDE upregulates ATAD2 expression via inhibiting miR-126-5p expression	Colorectal carcinoma	56
miR-126-5p	miRNA	-	miR-126-5p directly targets ATAD2 and downregulates its expression	Colorectal carcinoma	56
E2F1	transcription factor	+	E2F1 directly target ATAD2 by binding to and activating the ATAD2 promoter.	Breast cancer	64
AR	Androgen receptor	+	Androgens activates ATAD2 via directly interaction of AR with an ARBS within the gene	Prostate cancer	65
HIF1α	transcription factor	+	HIF1α as the transcription factor mediating upregulation of ATAD2 in hypoxic SCCs	Stomach cancer	59
MYBL2	Transcription factor	+	MYBL2 binds the CHR elements in the ATAD2 promoter to promote ATAD2 expression	Ovarian cancer	60
KDM8/JMJD5	Protein	+	KDM8 directly targets ATAD2 and up-regulates its expression	Prostate cancer	19
TRIM25	Protein	+	TRIM25 promoted ATAD2 expression in a dose-dependent and transcriptional-independent manner	Colorectal cancer	61
METTL3	Protein	+	METTL3 up-regulates ATAD2 expression, but the regulatory mechanism is unclear	Osteosarcoma	6
Derlin-1	Protein	+	Derlin-1 directly upregulates ATAD2 expression	Breast cancer	62
YAP1/TAZ	Protein	+	YAP1/TAZ enters the nucleus and binds to the promoter of ATAD2 to promote ATAD2 expression	Head and neck squamous cell carcinoma (HNSCC)	63

## Abbreviations

5α-dihydrotestosterone: 5α-DHT
Androgen receptor: AR
ATPase family AAA domain-containing protein 2: ATAD2
Breast cancer: BC
Bromine domain: BRD
cAMP-responsive element-binding protein: CBP/p300
C-Jun N-terminal kinase 1/2: JNK1/2
Colorectal cancer: CRC
C-terminal domain: CTD
Cyclin-dependent protein kinases: CDKs
DNA damage and repair: DDR
E2F transcription factor 1: E2F1
E2F transcription factor 2: E2F2
E2F transcription factor 3: E2F3
Enhancer of zeste homologue 2: EZH2
Epithelial-mesenchymal transition: EMT
Esophageal squamous cell carcinoma: ESCC
Estrogen receptor α: ERα
Extracellular signal-regulated kinase 1/2: ERK1/2
Fluorodeoxyglucose: FDG
Gastric cancer: GC
Glucose transporter type 1: GLUT1
Head and neck squamous cell carcinoma: HNSCC
Hedgehog: HH
Hepatocellular carcinoma: HCC
Hexokinase 2: HK2
HIF1α binding site: HBS
Histone deacetylases 2: HDAC2
Homologous recombination repair: HRR
Host cytokine 1: HCF-1
Hypoxia-inducible factor 1: HIF1
Long noncoding RNAs: lncRNAs
Lysine acetylation: KAc
Mammalian target of rapamycin: mTOR
MAP Kinase Kinase 3: MKK3/6
Methyltransferase-like 3: METTL3
MicroRNAs: miRNAs
Mitogen-activated protein kinase: MAPK
Mixed lineage protein-1: MLL1
MYC-interacting zinc finger protein 1: MIZ1
N-terminal acidic domain: NTD
Nuclear paraspeckle assembly transcript 1_2: NEAT1_2
Oral squamous cell carcinoma: OSCC
Patched 1: PTCH1
Phosphatidylinositol 3 kinase: PI3K
Retinoblastoma tumor suppressor protein: Rb
RNA-induced silencing complex: RISC
Smoothened: SMO
Stomach cancer cells: SCC
